# Supplementary material for: From Natural Xanthones to Synthetic C-1 Aminated 3,4-Dioxygenated Xanthones as Optimized Antifouling Agents
Source: Mar Drugs. 2021 Nov 13;19(11):638. doi: 10.3390/md19110638 (PMC8618441; doi:10.3390/md19110638)
Supplement: Supplementary file 1 [file marinedrugs-19-00638-s001.zip › marinedrugs-1440504-supplementary.pdf]

*Article*

# From natural xanthones to synthetic C-1 aminated 3,4-dioxygenated xanthones as optimized antifouling agents

**Diana I. S. P. Resende [1,2,#], Joana R. Almeida [1,#], Sandra Pereira [1,3], Alexandre Campos [1], Agostinho Lemos [2], Jeffrey E. Plowman [4], Ancy Thomas [4], Stefan Clerens [4,5,6], Vitor Vasconcelos [1,3], Madalena Pinto [1,2], Marta Correia-da-Silva [1,2,*] and Emília Sousa [1,2]**

[1] CIIMAR/CIMAR - Interdisciplinary Centre of Marine and Environmental Research, University of Porto, Terminal de Cruzeiros do Porto de Leixões, Avenida General, Norton de Matos S/N, 4450-208, Matosinhos, Portugal; joana.reis.almeida@gmail.com, sandra.c.pereira28@gmail.com, amoclclix@gmail.com, vmvascon@fc.up.pt
[2] Laboratory of Organic and Pharmaceutical Chemistry, Department of Chemical Sciences, Faculty of Pharmacy, University of Porto, Rua Jorge Viterbo Ferreira, 228, 4050-313, Porto, Portugal; dresende@ff.up.pt, up201002662@ff.up.pt; madalenakijjoa@gmail.com, esousaf.up.pt
[3] Department of Biology, Faculty of Sciences, University of Porto, Rua do Campo Alegre, 4069-007, Porto, Portugal
[4] AgResearch Ltd, 1365 Springs Rd, Lincoln 7674, New Zealand; Jeff.Plowman@agresearch.co.nz; Ancy.Thomas@agresearch.co.nz; Stefan.Clerens@agresearch.co.nz
[5] Biomolecular Interaction Centre, University of Canterbury, Christchurch, New Zealand
[6] Riddet Institute, Massey University, Palmerston North, New Zealand
*Correspondence: m_correiadasilva@ff.up.pt; Tel.: +351-220428689
# Both authors contributed equally to this work.

## Content

Figure S1. Concentration-response of antibacterial activity of the promising AF compounds 6 and 16 towards Roseobacter litoralis. (B: DMSO control; (C) penicillin-streptomycin-neomycin stabilized solution was used as positive control...................... 2

Table S1: Relative abundance values of proteins differentially expressed between control and group exposed to compound 21, and statistical results (mixed model ANOVA, P-value <0.05)........................................................................................ 3

Table S2: Relative abundance values of proteins differentially expressed between control and group exposed to compound 23, and statistical results (mixed model ANOVA, P-value <0.05)........................................................................................ 4

Table S3: Functional analysis and enrichment of differentially expressed proteins identified in mussel plantigrade larvae. Only the GO category Biological Process is represented in the analysis. GO = gene ontology term; term_size = number of gene sequences related to the GO term in the database; effective_domain_size = total number of gene sequences in the database. ................................................................. 6

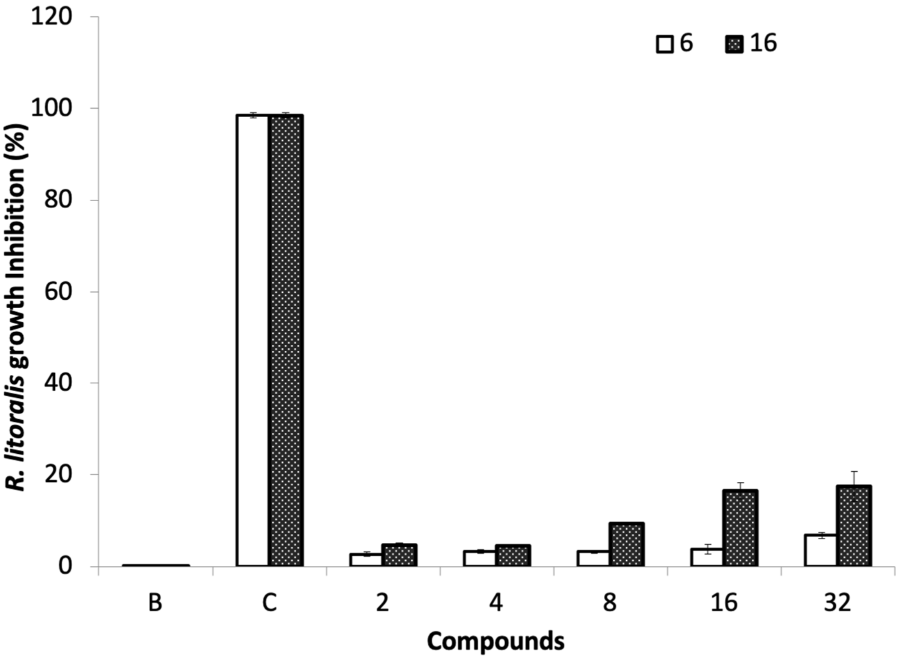


*Figure S1. Concentration-response of antibacterial activity of the promising AF compounds 6 and 16 towards Roseobacter litoralis. (B: DMSO control; (C) penicillin-streptomycin-neomycin stabilized solution was used as positive control.*

*Table S1: Relative abundance values of proteins differentially expressed between control and group exposed to compound 21, and statistical results (mixed model ANOVA, P-value <0.05)*

| | Mean of $log_{10}(x+1)$-transformed values | | | | |
|---|---|---|---|---|---|
| Protein ID | Control | compound 21 | P-value | Description | Accession |
| 2 | 4,6084 | 4,4047 | 0,024 | beta-actin [Meretrix meretrix] | AEK81538.1 |
| 9 | 4,2412 | 4,0767 | 0,036 | retrograde protein of 51 kDa-like isoform X4 [Crassostrea virginica] | XP_022320110.1 |
| 7 | 4,3863 | 4,0840 | 0,000 | PREDICTED: tubulin beta chain [Priapulus caudatus] | XP_014664190.1 |
| 11 | 4,3091 | 4,1307 | 0,013 | RecName: Full=Tropomyosin | Q25457.1 |
| 20 | 3,5028 | 3,2405 | 0,001 | sodium/potassium-transporting ATPase subunit alpha-like [Crassostrea virginica] | XP_022323941.1 |
| 15 | 4,2940 | 4,0813 | 0,014 | calponin-like protein [Mytilus galloprovincialis] | BAB60813.1 |
| 30 | 4,1447 | 3,9373 | 0,003 | elongation factor 1 alpha [Mytilus galloprovincialis] | BAD35019.1 |
| 61 | 4,7393 | 4,5857 | 0,022 | histone H2B [Mytilus edulis] | CAD37816.1 |
| 52 | 4,9618 | 4,7927 | 0,021 | PREDICTED: histone H4 [Equus caballus] | XP_014590042.1 |
| 42 | 3,8546 | 3,6500 | 0,001 | PREDICTED: 14-3-3 protein zeta isoform X1 [Hyalella azteca] | XP_018019106.1 |
| 122 | 3,8424 | 3,4942 | 0,000 | cyclophilin A isoform 2 [Ruditapes philippinarum] | ACU83230.1 |
| 5 | 3,8240 | 3,5656 | 0,003 | pedal retractor muscle myosin heavy chain partial [Mytilus galloprovincialis] | CAB64663.1 |
| 18 | 3,7831 | 3,5213 | 0,001 | mitochondrial H+ ATPase a subunit [Pinctada fucata] | ABJ51956.1 |
| 49 | 3,7566 | 3,5304 | 0,005 | myosin heavy chain isoform N [Anopheles sinensis] | KFB49247.1 |
| 51 | 4,0110 | 3,7157 | 0,000 | arginine kinase-like protein-1 [Mytilus coruscus] | AKS48144.1 |
| 109 | 3,2714 | 2,9889 | 0,007 | RNA-binding protein [Pinctada fucata] | ABP04054.1 |
| 35 | 3,8137 | 3,3956 | 0,000 | glyceraldehyde-3-phosphate dehydrogenase partial [Mytilus galloprovincialis] | AIK22450.1 |
| 34 | 3,7198 | 3,3952 | 0,001 | pedal retractor muscle myosin heavy chain partial [Mytilus galloprovincialis] | CAB64663.1 |
| 95 | 4,2033 | 3,9771 | 0,000 | histone H1 [Venerupis corrugata] | ABV25915.1 |
| 116 | 3,6801 | 3,3590 | 0,001 | meprin A subunit alpha-like [Mizuhopecten yessoensis] | XP_021356192.1 |
| 115 | 3,4571 | 3,2421 | 0,025 | PREDICTED: peroxiredoxin-6-like [Amphimedon queenslandica] | XP_003388603.1 |
| 17 | 3,8551 | 3,6858 | 0,028 | pedal retractor muscle myosin heavy chain partial [Mytilus galloprovincialis] | CAB64663.1 |
| 82 | 3,5652 | 3,2784 | 0,000 | elongation factor 2-like [Crassostrea virginica] | XP_022335104.1 |
| 40 | 3,4352 | 3,2845 | 0,032 | PREDICTED: alpha-actinin sarcomeric isoform X2 [Crassostrea gigas] | XP_011425356.1 |
| 23 | 3,5226 | 3,1429 | 0,000 | byssal HSP-like protein 1 [Mytilus coruscus] | ANN45953.1 |
| 14 | 3,8376 | 3,5327 | 0,000 | procollagen-proline dioxygenase beta subunit [Mytilus galloprovincialis] | AFM30917.1 |
| 64 | 3,7002 | 3,3308 | 0,000 | meprin A subunit alpha-like [Mizuhopecten yessoensis] | XP_021356192.1 |
| 75 | 3,3452 | 2,9697 | 0,000 | PREDICTED: adenosylhomocysteinase A [Crassostrea gigas] | XP_011412005.1 |
| 66 | 3,4001 | 3,2653 | 0,022 | uncharacterized protein LOC111134038 isoform X1 [Crassostrea virginica] | XP_022338516.1 |
| 91 | 3,4908 | 3,1630 | 0,000 | 40S ribosomal protein S14 [Mizuhopecten yessoensis] | XP_021365459.1 |
| 24 | 3,6329 | 3,3272 | 0,002 | heat shock cognate 71 [Mytilus galloprovincialis] | CAH04109.1 |
| 55 | 3,4341 | 3,1075 | 0,001 | ribosomal protein S8e partial [Mytilus trossulus] | ALX27203.1 |
| 93 | 3,5417 | 3,0424 | 0,000 | PREDICTED: calreticulin-like [Lingula anatina] | XP_013397065.1 |
| 118 | 3,4992 | 3,2132 | 0,001 | cytosolic malate dehydrogenase [Mytilus galloprovincialis] | AAZ79367.1 |
| 77 | 3,3442 | 3,0985 | 0,004 | PREDICTED: plastin-3 [Crassostrea gigas] | XP_011415779.2 |
| 10 | 3,4426 | 3,1885 | 0,007 | filamin-like protein-1 [Mytilus coruscus] | AKS48135.1 |
| 50 | 3,4594 | 3,1153 | 0,001 | PREDICTED: 14-3-3 protein epsilon isoform X1 [Ictalurus punctatus] | XP_017346722.1 |
| 21 | 3,5785 | 3,2910 | 0,001 | heat shock protein 90 [Mytilus galloprovincialis] | CAJ85741.1 |
| 80 | 3,2985 | 3,0979 | 0,012 | PREDICTED: mitochondria-eating protein isoform X2 [Crassostrea gigas] | XP_011417414.1 |
| 70 | 3,2833 | 3,0558 | 0,007 | aldehyde dehydrogenase mitochondrial-like [Mizuhopecten yessoensis] | XP_021346041.1 |
| 78 | 3,5655 | 3,1869 | 0,000 | predicted protein [methanotrophic endosymbiont of Bathymodiolus azoricus (Menez Gwen)] | SCN46548.1 |
| 39 | 3,5150 | 3,1624 | 0,000 | uncharacterized protein LOC111107057 isoform X2 [Crassostrea virginica] | XP_022297716.1 |
| 103 | 3,7262 | 3,3918 | 0,004 | mammalian ependymin-related protein 1-like [Mizuhopecten yessoensis] | XP_021339375.1 |
| 92 | 3,6673 | 3,3258 | 0,000 | voltage-dependent anion channel 2-like protein [Haliotis diversicolor] | ADI56517.1 |
| 48 | 3,5458 | 3,0795 | 0,001 | ribosomal protein [Mytilus galloprovincialis] | CAQ63458.1 |
| 99 | 3,1268 | 2,9581 | 0,011 | twitchin-like protein-1 [Mytilus coruscus] | AKS48140.1 |
| 106 | 3,4244 | 3,1177 | 0,000 | hypothetical protein LOTGIDRAFT_235900 [Lottia gigantea] | XP_009064140.1 |
| 74 | 3,4207 | 3,2090 | 0,008 | fructose-bisphosphate aldolase-like [Crassostrea virginica] | XP_022340550.1 |
| 101 | 3,2671 | 3,0932 | 0,012 | lamin Dm0-like [Mizuhopecten yessoensis] | XP_021357386.1 |
| 87 | 3,0554 | 2,6943 | 0,001 | adenylyl cyclase-associated protein 1-like isoform X3 [Crassostrea virginica] | XP_022334187.1 |
| 56 | 3,2693 | 2,9994 | 0,003 | myosin heavy chain non-muscle-like isoform X4 [Mizuhopecten yessoensis] | XP_021350592.1 |
| 19 | 3,7421 | 3,4603 | 0,000 | ATP synthase subunit beta mitochondrial [Mizuhopecten yessoensis] | XP_021356377.1 |
| 67 | 3,9791 | 3,8292 | 0,030 | histone H2A.Z.2 [Mytilus galloprovincialis] | APR72375.1 |
| 119 | 3,2063 | 2,9276 | 0,007 | Phosphoenolpyruvate carboxykinase [GTP] [Crassostrea gigas] | EKC27095.1 |
| 100 | 3,2320 | 2,9014 | 0,001 | elongation factor 1-gamma-like [Crassostrea virginica] | XP_022332183.1 |
| 90 | 3,2416 | 2,8597 | 0,004 | citrate synthase mitochondrial-like [Mizuhopecten yessoensis] | XP_021357329.1 |
| 105 | 3,0641 | 2,7813 | 0,034 | flotillin-1-like isoform X1 [Mizuhopecten yessoensis] | XP_021361554.1 |
| 12 | 3,8573 | 3,5600 | 0,003 | tubulin alpha-1A chain [Mizuhopecten yessoensis] | XP_021370666.1 |
| 123 | 3,6254 | 3,4409 | 0,002 | ubiquitin-40S ribosomal protein S27a [Crassostrea gigas] | NP_001292281.1 |
| 53 | 3,1497 | 2,8956 | 0,006 | tubulin beta chain-like [Crassostrea gigas] | NP_001292292.1 |
| 144 | 3,8766 | 3,5641 | 0,004 | unknown | |
| 86 | 2,7963 | 2,9463 | 0,047 | PREDICTED: laminin subunit gamma-1 [Crassostrea gigas] | XP_011419247.1 |
| 249 | 3,1630 | 2,9970 | 0,012 | uncharacterized protein ZK1073.1-like isoform X4 [Crassostrea virginica] | XP_022335676.1 |
| 152 | 3,3320 | 3,0606 | 0,001 | PREDICTED: 40S ribosomal protein S3 [Crassostrea gigas] | XP_011438308.1 |
| 54 | 3,4558 | 3,1287 | 0,001 | isocitrate dehydrogenase [Mytilus galloprovincialis] | AFI56365.1 |
| 114 | 3,4954 | 3,1848 | 0,001 | uncharacterized protein LOC110457451 isoform X2 [Mizuhopecten yessoensis] | XP_021364413.1 |
| 200 | 3,0633 | 2,8996 | 0,006 | uncharacterized protein LOC111111285 isoform X1 [Crassostrea virginica] | XP_022303867.1 |
| 113 | 3,2925 | 3,0384 | 0,004 | PREDICTED: 60S ribosomal protein L11 [Biomphalaria glabrata] | XP_013068480.1 |
| 162 | 2,8043 | 2,4909 | 0,001 | uncharacterized protein LOC110465734 isoform X2 [Mizuhopecten yessoensis] | XP_021377443.1 |
| 128 | 3,3487 | 3,0454 | 0,001 | cathepsin D [Pteria penguin] | AEI58895.1 |
| 143 | 3,2502 | 2,7554 | 0,000 | 40S ribosomal protein S25-like [Mizuhopecten yessoensis] | XP_021370373.1 |
| 71 | 2,9901 | 2,7168 | 0,015 | dolichyl-diphosphooligosaccharide--protein glycosyltransferase subunit 1-like [Mizuhopecten ye | XP_021341263.1 |
| 108 | 2,7362 | 2,4491 | 0,037 | hemicentin-1-like partial [Mizuhopecten yessoensis] | XP_021352826.1 |
| 26 | 3,5214 | 3,1616 | 0,000 | protein disulfide-isomerase A3-like [Crassostrea virginica] | XP_022329914.1 |
| 111 | 3,1931 | 3,0007 | 0,046 | small heat shock protein 22 [Mytilus galloprovincialis] | AEP02967.1 |
| 137 | 3,4747 | 3,0260 | 0,000 | PREDICTED: peptidyl-prolyl cis-trans isomerase [Crassostrea gigas] | XP_011444269.1 |
| 156 | 3,0107 | 2,6969 | 0,005 | PREDICTED: major vault protein [Crassostrea gigas] | XP_011432755.1 |
| 132 | 3,4018 | 3,1217 | 0,001 | malate dehydrogenase precursor [Nucella lapillus] | AAF27650.1 |
| 102 | 2,9632 | 2,7625 | 0,017 | twitchin [Mytilus galloprovincialis] | BAC00784.1 |
| 160 | 3,2879 | 2,9446 | 0,000 | PREDICTED: protein SET [Crassostrea gigas] | XP_011443579.1 |
| 3678 | 2,6600 | 2,2732 | 0,000 | PREDICTED: tektin-4 [Crassostrea gigas] | XP_011416098.1 |
| 7957 | 3,0717 | 2,6923 | 0,006 | 40S ribosomal protein S4-like [Crassostrea virginica] | XP_022290789.1 |
| 158 | 3,1664 | 2,8698 | 0,000 | LOW QUALITY PROTEIN: cilia- and flagella-associated protein 161-like [Crassostrea virginica] | XP_022332848.1 |
| 169 | 3,0207 | 2,5181 | 0,035 | dolichyl-diphosphooligosaccharide--protein glycosyltransferase 48 kDa subunit-like [Mizuhopec | XP_021367901.1 |
| 107 | 3,2327 | 2,9822 | 0,002 | PREDICTED: nucleolin-like partial [Biomphalaria glabrata] | XP_013075907.1 |
| 133 | 2,9688 | 2,2429 | 0,043 | PREDICTED: 60S ribosomal protein L22-like [Crassostrea gigas] | XP_011451810.1 |
| 154 | 3,2849 | 2,7622 | 0,000 | glutathione S-transferase sigma 2 [Mytilus galloprovincialis] | AFQ35984.1 |
| 110 | 2,9092 | 2,5755 | 0,001 | PREDICTED: radixin isoform X3 [Crassostrea gigas] | XP_011453118.1 |
| 141 | 3,2149 | 2,1599 | 0,000 | PREDICTED: uncharacterized protein LOC105329621 isoform X2 [Crassostrea gigas] | XP_011429241.1 |
| 258 | 1,9741 | 0,0000 | 0,010 | uncharacterized protein LOC110464458 isoform X12 [Mizuhopecten yessoensis] | XP_021375365.1 |

| | | | | | |
|---|---|---|---|---|---|
| 76 | 2,9921 | 2,7453 | 0,025 | histone variant H2A.X [Mytilus galloprovincialis] | AEH58057.1 |
| 147 | 3,2247 | 2,8937 | 0,026 | glutathione S-transferase sigma 3 [Mytilus galloprovincialis] | AFQ35985.1 |
| 171 | 3,1081 | 2,8633 | 0,019 | pedal retractor muscle myosin heavy chain partial [Mytilus galloprovincialis] | CAB64663.1 |
| 151 | 2,4658 | 0,6680 | 0,026 | hypothetical protein HELRODRAFT_108067 partial [Helobdella robusta] | XP_009028970.1 |
| 124 | 3,2071 | 2,9931 | 0,005 | RecName: Full=Calmodulin; Short=CaM | P27166.2 |
| 230 | 3,3076 | 3,0505 | 0,001 | hypothetical protein AM593_04533 partial [Mytilus galloprovincialis] | OPL33555.1 |
| 220 | 2,3324 | 2,0789 | 0,002 | T-complex protein 1 subunit eta-like [Crassostrea virginica] | XP_022288360.1 |
| 121 | 2,7496 | 2,5490 | 0,026 | filamin-like protein-1 [Mytilus coruscus] | AKS48135.1 |
| 3693 | 1,4823 | 0,0000 | 0,031 | clathrin heavy chain 1 isoform X1 [Mizuhopecten yessoensis] | XP_021354511.1 |
| 148 | 3,2640 | 2,9852 | 0,001 | hypothetical protein AM593_00614 partial [Mytilus galloprovincialis] | OPL20910.1 |
| 256 | 3,0412 | 2,7527 | 0,003 | 60S ribosomal protein L7-like [Crassostrea virginica] | XP_022339290.1 |
| 44 | 3,4977 | 3,2103 | 0,001 | hypothetical protein HELRODRAFT_186143 [Helobdella robusta] | XP_009029528.1 |
| 264 | 2,8252 | 2,5685 | 0,001 | unknown | |
| 197 | 2,8570 | 2,5373 | 0,000 | PREDICTED: enolase-phosphatase E1 [Crassostrea gigas] | XP_011428851.1 |
| 7960 | 2,9838 | 2,6435 | 0,000 | heterogeneous nuclear ribonucleoprotein D-like isoform X3 [Mizuhopecten yessoensis] | XP_021369725.1 |
| 1149 | 3,4198 | 3,1312 | 0,007 | PREDICTED: centlein [Crassostrea gigas] | XP_019925172.1 |
| 7956 | 3,0254 | 2,8079 | 0,005 | heterogeneous nuclear ribonucleoprotein K-like isoform X7 [Mizuhopecten yessoensis] | XP_021358952.1 |
| 202 | 3,0430 | 2,7488 | 0,000 | 60S ribosomal protein L23 [Mizuhopecten yessoensis] | XP_021373043.1 |
| 396 | 2,8174 | 2,4655 | 0,000 | TCTP [Mytilus galloprovincialis] | AEV41412.1 |
| 209 | 2,9780 | 2,6400 | 0,000 | 60S ribosomal protein L9 [Mizuhopecten yessoensis] | OWF37275.1 |
| 89 | 3,0866 | 2,8297 | 0,012 | putative calmodulin variant 1 [Taeniopygia guttata] | ACH46222.1 |
| 307 | 3,3333 | 3,5415 | 0,019 | unknown | |
| 342 | 2,8087 | 2,5753 | 0,001 | tektin-2-like [Crassostrea virginica] | XP_022314135.1 |
| 255 | 2,9807 | 2,6094 | 0,000 | 60S ribosomal protein L12-like [Crassostrea virginica] | XP_022314628.1 |
| 201 | 3,0852 | 2,4743 | 0,001 | unknown | |
| 176 | 2,7170 | 2,5248 | 0,005 | PREDICTED: 60S ribosomal protein L18-like [Lingula anatina] | XP_013398405.1 |
| 206 | 2,9052 | 2,7401 | 0,016 | PREDICTED: guanine nucleotide-binding protein subunit beta [Crassostrea gigas] | XP_011419294.1 |
| 226 | 3,0508 | 2,8436 | 0,002 | 40S ribosomal protein S26-like [Mizuhopecten yessoensis] | XP_021375377.1 |
| 216 | 2,8254 | 2,5486 | 0,002 | glutamate dehydrogenase mitochondrial-like [Crassostrea virginica] | XP_022314664.1 |
| 3688 | 2,8692 | 2,5381 | 0,001 | hypothetical protein LOTGIDRAFT_214936 [Lottia gigantea] | XP_009053796.1 |
| 205 | 3,0505 | 2,6489 | 0,000 | PREDICTED: 40S ribosomal protein S13 [Crassostrea gigas] | XP_011441368.1 |
| 164 | 3,0233 | 2,7728 | 0,001 | PREDICTED: filamin-A isoform X3 [Crassostrea gigas] | XP_019922596.1 |
| 233 | 2,7204 | 2,4949 | 0,006 | PREDICTED: nucleoredoxin [Strongylocentrotus purpuratus] | XP_011682183.1 |
| 368 | 2,7200 | 2,6087 | 0,027 | T-complex protein 1 subunit beta-like [Crassostrea virginica] | XP_022286485.1 |
| 159 | 2,7381 | 2,4235 | 0,004 | 60S ribosomal protein L7a-like [Mizuhopecten yessoensis] | XP_021369622.1 |
| 211 | 2,7897 | 2,4614 | 0,006 | calmodulin-like isoform X1 [Crassostrea virginica] | XP_022303657.1 |
| 217 | 2,5650 | 2,3167 | 0,004 | insulin-like growth factor 2 mRNA-binding protein 2 isoform X1 [Crassostrea virginica] | XP_022305782.1 |
| 3692 | 2,9455 | 2,7034 | 0,001 | 60S ribosomal protein L10-like [Crassostrea virginica] | XP_022315909.1 |
| 235 | 2,5877 | 2,3636 | 0,010 | PREDICTED: alcohol dehydrogenase class-3-like [Lingula anatina] | XP_013411149.1 |
| 186 | 3,0129 | 2,7163 | 0,002 | PREDICTED: enolase-like [Crassostrea gigas] | XP_011436228.1 |
| 168 | 2,7762 | 2,4933 | 0,025 | eukaryotic initiation factor 4A-I-like [Crassostrea virginica] | XP_022288944.1 |
| 117 | 2,5597 | 2,3111 | 0,012 | LOW QUALITY PROTEIN: annexin A4-like [Crassostrea virginica] | XP_022316814.1 |
| 260 | 2,5457 | 2,1316 | 0,001 | cytochrome c oxidase subunit IV partial [Mytilus edulis] | ACA23124.1 |
| 104 | 2,6860 | 2,3371 | 0,001 | Ras-related protein Rab-1A [Haliotis discus discus] | ABO26625.1 |
| 251 | 2,5156 | 2,1923 | 0,001 | PREDICTED: protein disulfide-isomerase A6 [Crassostrea gigas] | XP_011446200.1 |
| 5888 | 2,4391 | 2,2288 | 0,006 | testis-specific gene 10 protein-like isoform X2 [Mizuhopecten yessoensis] | XP_021348266.1 |
| 155 | 2,6237 | 2,1962 | 0,000 | PREDICTED: nucleoside diphosphate kinase A [Crassostrea gigas] | XP_011449577.1 |
| 227 | 2,4564 | 1,4509 | 0,048 | PREDICTED: cilia- and flagella-associated protein 52 [Crassostrea gigas] | XP_011416101.1 |
| 59 | 2,4320 | 2,1272 | 0,010 | RecName: Full=Histone H2A | Q6WV66.3 |
| 297 | 2,4459 | 2,1472 | 0,001 | PREDICTED: limbin-like isoform X2 [Crassostrea gigas] | XP_011443052.1 |
| 7955 | 3,0369 | 2,8432 | 0,038 | LKD-rich protein-1 [Mytilus galloprovincialis] | AKS48185.1 |
| 3689 | 2,7744 | 2,5032 | 0,002 | pyruvate kinase PKM-like isoform X6 [Crassostrea virginica] | XP_022311729.1 |
| 271 | 2,7173 | 2,3857 | 0,002 | hypothetical protein AM593_10055 partial [Mytilus galloprovincialis] | OPL21226.1 |
| 7958 | 2,5175 | 2,2083 | 0,005 | PREDICTED: aspartate aminotransferase cytoplasmic [Crassostrea gigas] | XP_011430094.1 |
| 244 | 2,4733 | 2,1115 | 0,002 | ras [Mytilus edulis] | AAT81171.1 |
| 214 | 2,5957 | 2,3405 | 0,044 | 6-phosphogluconate dehydrogenase decarboxylating-like [Crassostrea virginica] | XP_022343934.1 |
| 231 | 2,5772 | 2,2819 | 0,000 | PREDICTED: cytosolic non-specific dipeptidase isoform X1 [Crassostrea gigas] | XP_019928389.1 |
| 222 | 2,5655 | 2,1826 | 0,000 | annexin B9-like [Crassostrea virginica] | XP_022317867.1 |
| 263 | 2,7963 | 2,5108 | 0,016 | major vault protein-like [Crassostrea virginica] | XP_022342755.1 |
| 268 | 2,8869 | 2,6757 | 0,003 | 14-3-3 protein homolog 2-like [Crassostrea virginica] | XP_022343021.1 |
| 5863 | 2,4225 | 0,8739 | 0,032 | PREDICTED: transketolase-like protein 2 isoform X1 [Crassostrea gigas] | XP_011427761.1 |
| 131 | 2,5765 | 1,8677 | 0,001 | 60S ribosomal protein L10a-2-like [Mizuhopecten yessoensis] | XP_021379854.1 |
| 312 | 2,8012 | 2,6169 | 0,020 | PREDICTED: troponin T-like [Biomphalaria glabrata] | XP_013095195.1 |
| 5871 | 2,4403 | 1,7548 | 0,011 | radial spoke head protein 4 homolog A-like [Mizuhopecten yessoensis] | XP_021370563.1 |
| 85 | 2,9117 | 2,6880 | 0,022 | filamin-like protein-3 partial [Mytilus coruscus] | AKS48150.1 |
| 3694 | 2,3432 | 1,6698 | 0,008 | radial spoke head protein 9 homolog [Mizuhopecten yessoensis] | XP_021363337.1 |

*Table S2: Relative abundance values of proteins differentially expressed between control and group exposed to compound 23, and statistical results (mixed model ANOVA, P-value <0.05)*

| | Mean of $log_{10}(x+1)$-transformed values | | | | |
|---|---|---|---|---|---|
| Protein ID | Control | compound 23 | P-value (a) | protein description | Accession |
| 48 | 3,545820403 | 3,12534832 | 0,000 | ribosomal protein [Mytilus galloprovincialis] | CAQ63458.1 |
| 220 | 2,332414357 | 2,115023251 | 0,000 | T-complex protein 1 subunit eta-like [Crassostrea virginica] | XP_022288360.1 |
| 75 | 3,345207927 | 3,056096356 | 0,000 | PREDICTED: adenosylhomocysteinase A [Crassostrea gigas] | XP_011412005.1 |
| 151 | 2,465766659 | 0 | 0,000 | hypothetical protein HELRODRAFT_108067 partial [Helobdella robusta] | XP_009028970.1 |
| 200 | 3,063263125 | 2,893022785 | 0,000 | uncharacterized protein LOC111111285 isoform X1 [Crassostrea virginica] | XP_022303867.1 |
| 14 | 3,83764251 | 3,605742532 | 0,000 | procollagen-proline dioxygenase beta subunit [Mytilus galloprovincialis] | AFM30917.1 |
| 128 | 3,34871817 | 3,075801472 | 0,001 | cathepsin D [Pteria penguin] | AEI58895.1 |
| 147 | 3,224718342 | 2,737205761 | 0,001 | glutathione S-transferase sigma 3 [Mytilus galloprovincialis] | AFQ35985.1 |
| 312 | 2,801211375 | 2,469386138 | 0,001 | PREDICTED: troponin T-like [Biomphalaria glabrata] | XP_013095195.1 |
| 124 | 3,207143385 | 3,00324297 | 0,001 | RecName: Full=Calmodulin; Short=CaM | P27166.2 |
| 93 | 3,541707496 | 3,228150096 | 0,001 | PREDICTED: calreticulin-like [Lingula anatina] | XP_013397065.1 |
| 396 | 2,817361952 | 2,554494681 | 0,001 | TCTP [Mytilus galloprovincialis] | AEV41412.1 |
| 204 | 3,233751495 | 2,998238284 | 0,001 | PREDICTED: 40S ribosomal protein SA [Crassostrea gigas] | XP_011438171.1 |
| 123 | 3,625407615 | 3,445274517 | 0,002 | ubiquitin-40S ribosomal protein S27a [Crassostrea gigas] | NP_001292281.1 |
| 209 | 2,977999002 | 2,756523602 | 0,002 | 60S ribosomal protein L9 [Mizuhopecten yessoensis] | OWF37275.1 |
| 39 | 3,515027408 | 3,273928967 | 0,002 | uncharacterized protein LOC111107057 isoform X2 [Crassostrea virginica] | XP_022297716.1 |
| 164 | 3,023345072 | 2,790851915 | 0,002 | PREDICTED: filamin-A isoform X3 [Crassostrea gigas] | XP_019922596.1 |
| 7957 | 3,071678554 | 2,708424086 | 0,002 | 40S ribosomal protein S4-like [Crassostrea virginica] | XP_022290789.1 |
| 19 | 3,742052665 | 3,5100083 | 0,002 | ATP synthase subunit beta mitochondrial [Mizuhopecten yessoensis] | XP_021356377.1 |
| 211 | 2,789723988 | 2,370858204 | 0,002 | calmodulin-like isoform X1 [Crassostrea virginica] | XP_022303657.1 |
| 71 | 2,990050751 | 2,725199804 | 0,002 | dolichyl-diphosphooligosaccharide--protein glycosyltransferase subunit 1-like [Mizuhope | XP_021341263.1 |

| | | | | | |
|---|---|---|---|---|---|
| 118 | 3,499157843 | 3,248432026 | 0,003 | cytosolic malate dehydrogenase [Mytilus galloprovincialis] | AAZ79367.1 |
| 11 | 4,309132629 | 4,113950451 | 0,003 | RecName: Full=Tropomyosin | Q25457.1 |
| 116 | 3,680084414 | 3,459045841 | 0,003 | meprin A subunit alpha-like [Mizuhopecten yessoensis] | XP_021356192.1 |
| 64 | 3,700154371 | 3,452822857 | 0,003 | meprin A subunit alpha-like [Mizuhopecten yessoensis] | XP_021356192.1 |
| 95 | 4,203265567 | 4,025946156 | 0,003 | histone H1 [Venerupis corrugata] | ABV25915.1 |
| 152 | 3,332009681 | 3,112969466 | 0,003 | PREDICTED: 40S ribosomal protein S3 [Crassostrea gigas] | XP_011438308.1 |
| 154 | 3,284865997 | 2,976084697 | 0,003 | glutathione S-transferase sigma 2 [Mytilus galloprovincialis] | AFQ35984.1 |
| 143 | 3,250182868 | 2,850487771 | 0,003 | 40S ribosomal protein S25-like [Mizuhopecten yessoensis] | XP_021370373.1 |
| 216 | 2,825359561 | 2,536664209 | 0,003 | glutamate dehydrogenase mitochondrial-like [Crassostrea virginica] | XP_022314664.1 |
| 107 | 3,232661416 | 3,009390822 | 0,003 | PREDICTED: nucleolin-like partial [Biomphalaria glabrata] | XP_013075907.1 |
| 104 | 2,686042679 | 2,428289828 | 0,003 | Ras-related protein Rab-1A [Haliotis discus discus] | ABO26625.1 |
| 121 | 2,749600201 | 2,525639385 | 0,003 | filamin-like protein-1 [Mytilus coruscus] | AKS48135.1 |
| 101 | 3,267113764 | 3,060893175 | 0,003 | lamin Dm0-like [Mizuhopecten yessoensis] | XP_021357386.1 |
| 18 | 3,783148688 | 3,583237842 | 0,003 | mitochondrial H+ ATPase a subunit [Pinctada fucata] | ABJ51956.1 |
| 7 | 4,386337293 | 4,161511387 | 0,003 | PREDICTED: tubulin beta chain [Priapulus caudatus] | XP_014664190.1 |
| 7960 | 2,983821018 | 2,703736298 | 0,003 | heterogeneous nuclear ribonucleoprotein D-like isoform X3 [Mizuhopecten yessoensis] | XP_021369725.1 |
| 92 | 3,667347121 | 3,408954738 | 0,003 | voltage-dependent anion channel 2-like protein [Haliotis diversicolor] | ADI56517.1 |
| 160 | 3,287916402 | 3,061218026 | 0,003 | PREDICTED: protein SET [Crassostrea gigas] | XP_011443579.1 |
| 156 | 3,010691413 | 2,820181491 | 0,003 | PREDICTED: major vault protein [Crassostrea gigas] | XP_011432755.1 |
| 51 | 4,011009496 | 3,768665654 | 0,004 | arginine kinase-like protein-1 [Mytilus coruscus] | AKS48144.1 |
| 34 | 3,719836167 | 3,465417116 | 0,004 | pedal retractor muscle myosin heavy chain partial [Mytilus galloprovincialis] | CAB64663.1 |
| 91 | 3,490803585 | 3,195927616 | 0,004 | 40S ribosomal protein S14 [Mizuhopecten yessoensis] | XP_021365459.1 |
| 255 | 2,980681499 | 2,752204267 | 0,004 | 60S ribosomal protein L12-like [Crassostrea virginica] | XP_022314628.1 |
| 106 | 3,424376599 | 3,176929458 | 0,004 | hypothetical protein LOTGIDRAFT_235900 [Lottia gigantea] | XP_009064140.1 |
| 2 | 4,608357188 | 4,331231822 | 0,004 | beta-actin [Meretrix meretrix] | AEK81538.1 |
| 23 | 3,522644814 | 3,253016622 | 0,004 | byssal HSP-like protein 1 [Mytilus coruscus] | ANN45953.1 |
| 66 | 3,400147735 | 3,24873257 | 0,004 | uncharacterized protein LOC111134038 isoform X1 [Crassostrea virginica] | XP_022338516.1 |
| 277 | 3,618155895 | 3,353228755 | 0,004 | PREDICTED: cilia- and flagella-associated protein 52 [Crassostrea gigas] | XP_011416101.1 |
| 82 | 3,56516495 | 3,338983112 | 0,004 | elongation factor 2-like [Crassostrea virginica] | XP_022335104.1 |
| 109 | 3,271415559 | 3,068852017 | 0,004 | RNA-binding protein [Pinctada fucata] | ABP04054.1 |
| 3678 | 2,660018778 | 2,383571891 | 0,004 | PREDICTED: tektin-4 [Crassostrea gigas] | XP_011416098.1 |
| 21 | 3,578521812 | 3,322667625 | 0,004 | heat shock protein 90 [Mytilus galloprovincialis] | CAJ85741.1 |
| 3 | 4,156373944 | 4,010703168 | 0,005 | actin adductor muscle [Crassostrea virginica] | XP_022325998.1 |
| 176 | 2,717024295 | 2,476587647 | 0,005 | PREDICTED: 60S ribosomal protein L18-like [Lingula anatina] | XP_013398405.1 |
| 100 | 3,23199652 | 2,965995553 | 0,005 | elongation factor 1-gamma-like [Crassostrea virginica] | XP_022332183.1 |
| 158 | 3,166385086 | 2,973392825 | 0,005 | LOW QUALITY PROTEIN: cilia- and flagella-associated protein 161-like [Crassostrea virginica | XP_022332848.1 |
| 202 | 3,043023332 | 2,836147023 | 0,005 | 60S ribosomal protein L23 [Mizuhopecten yessoensis] | XP_021373043.1 |
| 3688 | 2,869157615 | 2,61767721 | 0,005 | hypothetical protein LOTGIDRAFT_214936 [Lottia gigantea] | XP_009053796.1 |
| 55 | 3,434052278 | 3,15028305 | 0,005 | ribosomal protein S8e partial [Mytilus trossulus] | ALX27203.1 |
| 10 | 3,442643459 | 3,231196414 | 0,005 | filamin-like protein-1 [Mytilus coruscus] | AKS48135.1 |
| 122 | 3,842354494 | 3,616537791 | 0,005 | cyclophilin A isoform 2 [Ruditapes philippinarum] | ACU83230.1 |
| 162 | 2,804256103 | 2,590340589 | 0,005 | uncharacterized protein LOC110465734 isoform X2 [Mizuhopecten yessoensis] | XP_021377443.1 |
| 119 | 3,206300065 | 2,925458855 | 0,005 | Phosphoenolpyruvate carboxykinase [GTP] [Crassostrea gigas] | EKC27095.1 |
| 3689 | 2,774369561 | 2,540129165 | 0,005 | pyruvate kinase PKM-like isoform X6 [Crassostrea virginica] | XP_022311729.1 |
| 24 | 3,632923283 | 3,395332457 | 0,005 | heat shock cognate 71 [Mytilus galloprovincialis] | CAH04109.1 |
| 250 | 2,827231292 | 2,596015272 | 0,005 | triosephosphate isomerase-like [Crassostrea virginica] | XP_022336561.1 |
| 197 | 2,856985484 | 2,639983825 | 0,006 | PREDICTED: enolase-phosphatase E1 [Crassostrea gigas] | XP_011428851.1 |
| 52 | 4,961802114 | 4,698221583 | 0,006 | PREDICTED: histone H4 [Equus caballus] | XP_014590042.1 |
| 3681 | 3,17637219 | 2,982573632 | 0,006 | unknown | |
| 7956 | 3,025394087 | 2,823155779 | 0,006 | heterogeneous nuclear ribonucleoprotein K-like isoform X7 [Mizuhopecten yessoensis] | XP_021358952.1 |
| 305 | 2,573678629 | 2,324506715 | 0,006 | centrosome-associated protein 350-like isoform X1 [Mizuhopecten yessoensis] | XP_021341745.1 |
| 20 | 3,502750242 | 3,293095048 | 0,007 | sodium/potassium-transporting ATPase subunit alpha-like [Crassostrea virginica] | XP_022323941.1 |
| 77 | 3,344192149 | 3,110542141 | 0,007 | PREDICTED: plastin-3 [Crassostrea gigas] | XP_011415779.2 |
| 87 | 3,055405599 | 2,831648317 | 0,007 | adenylyl cyclase-associated protein 1-like isoform X3 [Crassostrea virginica] | XP_022334187.1 |
| 214 | 2,595710721 | 2,248472079 | 0,007 | 6-phosphogluconate dehydrogenase decarboxylating-like [Crassostrea virginica] | XP_022343934.1 |
| 3687 | 2,536499433 | 2,175207381 | 0,007 | hypothetical protein LOTGIDRAFT_187321 [Lottia gigantea] | XP_009050935.1 |
| 5 | 3,823973507 | 3,623290062 | 0,007 | pedal retractor muscle myosin heavy chain partial [Mytilus galloprovincialis] | CAB64663.1 |
| 56 | 3,269261884 | 2,947746249 | 0,007 | myosin heavy chain non-muscle-like isoform X4 [Mizuhopecten yessoensis] | XP_021350592.1 |
| 231 | 2,577154658 | 2,369180242 | 0,007 | PREDICTED: cytosolic non-specific dipeptidase isoform X1 [Crassostrea gigas] | XP_019928389.1 |
| 7958 | 2,517463899 | 2,266455214 | 0,007 | PREDICTED: aspartate aminotransferase cytoplasmic [Crassostrea gigas] | XP_011430094.1 |
| 264 | 2,825249311 | 2,641177745 | 0,007 | unknown | |
| 186 | 3,012938137 | 2,794362418 | 0,008 | PREDICTED: enolase-like [Crassostrea gigas] | XP_011436228.1 |
| 661 | 3,157541051 | 2,925461753 | 0,008 | hypothetical protein KP79_PYT11668 [Mizuhopecten yessoensis] | OWF55888.1 |
| 138 | 3,268109035 | 3,051029842 | 0,008 | hypothetical protein AM593_06933 partial [Mytilus galloprovincialis] | OPL33400.1 |
| 205 | 3,050502734 | 2,821032635 | 0,008 | PREDICTED: 40S ribosomal protein S13 [Crassostrea gigas] | XP_011441368.1 |
| 70 | 3,283349288 | 3,051638549 | 0,008 | aldehyde dehydrogenase mitochondrial-like [Mizuhopecten yessoensis] | XP_021346041.1 |
| 74 | 3,420666821 | 3,219288181 | 0,008 | fructose-bisphosphate aldolase-like [Crassostrea virginica] | XP_022340550.1 |
| 30 | 4,144743792 | 3,943383122 | 0,008 | elongation factor 1 alpha [Mytilus galloprovincialis] | BAD35019.1 |
| 103 | 3,726238911 | 3,491007989 | 0,009 | mammalian ependymin-related protein 1-like [Mizuhopecten yessoensis] | XP_021339375.1 |
| 244 | 2,473319478 | 2,185610261 | 0,009 | ras [Mytilus edulis] | AAT81171.1 |
| 35 | 3,813745702 | 3,559079375 | 0,009 | glyceraldehyde-3-phosphate dehydrogenase partial [Mytilus galloprovincialis] | AIK22450.1 |
| 3692 | 2,945486676 | 2,718155921 | 0,009 | 60S ribosomal protein L10-like [Crassostrea virginica] | XP_022315909.1 |
| 40 | 3,435168894 | 3,232487305 | 0,009 | PREDICTED: alpha-actinin sarcomeric isoform X2 [Crassostrea gigas] | XP_011425356.1 |
| 230 | 3,30764614 | 3,124902897 | 0,009 | hypothetical protein AM593_04533 partial [Mytilus galloprovincialis] | OPL33555.1 |
| 61 | 4,739270666 | 4,496380037 | 0,009 | histone H2B [Mytilus edulis] | CAD37816.1 |
| 131 | 2,576491164 | 2,155544421 | 0,009 | 60S ribosomal protein L10a-2-like [Mizuhopecten yessoensis] | XP_021379854.1 |
| 57 | 3,314710065 | 3,018562514 | 0,009 | PREDICTED: tektin-3-like isoform X1 [Crassostrea gigas] | XP_011450983.1 |
| 168 | 2,776191705 | 2,491219533 | 0,009 | eukaryotic initiation factor 4A-I-like [Crassostrea virginica] | XP_022288944.1 |
| 251 | 2,515581532 | 2,250862418 | 0,010 | PREDICTED: protein disulfide-isomerase A6 [Crassostrea gigas] | XP_011446200.1 |
| 42 | 3,854636586 | 3,682162944 | 0,010 | PREDICTED: 14-3-3 protein zeta isoform X1 [Hyalella azteca] | XP_018019106.1 |
| 159 | 2,738066745 | 2,428038805 | 0,010 | 60S ribosomal protein L7a-like [Mizuhopecten yessoensis] | XP_021369622.1 |
| 89 | 3,086601511 | 2,893283642 | 0,010 | putative calmodulin variant 1 [Taeniopygia guttata] | ACH46222.1 |
| 54 | 3,455775518 | 3,17412769 | 0,011 | isocitrate dehydrogenase [Mytilus galloprovincialis] | AFI56365.1 |
| 226 | 3,050841993 | 2,853249747 | 0,011 | 40S ribosomal protein S26-like [Mizuhopecten yessoensis] | XP_021375377.1 |
| 141 | 3,214948241 | 2,638804997 | 0,011 | PREDICTED: uncharacterized protein LOC105329621 isoform X2 [Crassostrea gigas] | XP_011429241.1 |
| 297 | 2,445944845 | 2,170092336 | 0,011 | PREDICTED: limbin-like isoform X2 [Crassostrea gigas] | XP_011443052.1 |
| 67 | 3,979066934 | 3,763014465 | 0,012 | histone H2A.Z.2 [Mytilus galloprovincialis] | APR72375.1 |

| | | | | |
|---|---|---|---|---|
| 171 | 3,108084961 | 2,872964259 | 0,012 pedal retractor muscle myosin heavy chain partial [Mytilus galloprovincialis] | CAB64663.1 |
| 290 | 3,597978116 | 3,986360585 | 0,012 multidrug resistance-associated protein 5-like [Crassostrea virginica] | XP_022324434.1 |
| 7955 | 3,036888247 | 2,769293038 | 0,012 LKD-rich protein-1 [Mytilus galloprovincialis] | AKS48185.1 |
| 256 | 3,0412126 | 2,731814666 | 0,012 60S ribosomal protein L7-like [Crassostrea virginica] | XP_022339290.1 |
| 50 | 3,4593682 | 3,207952064 | 0,013 PREDICTED: 14-3-3 protein epsilon isoform X1 [Ictalurus punctatus] | XP_017346722.1 |
| 132 | 3,401788602 | 3,182253001 | 0,013 malate dehydrogenase precursor [Nucella lapillus] | AAF27650.1 |
| 110 | 2,909183169 | 2,667474306 | 0,013 PREDICTED: radixin isoform X3 [Crassostrea gigas] | XP_011453118.1 |
| 148 | 3,264003471 | 3,054397116 | 0,013 hypothetical protein AM593_00614 partial [Mytilus galloprovincialis] | OPL20910.1 |
| 114 | 3,49538502 | 3,264693653 | 0,014 uncharacterized protein LOC110457451 isoform X2 [Mizuhopecten yessoensis] | XP_021364413.1 |
| 201 | 3,085176892 | 2,825275593 | 0,014 unknown | No_Hits |
| 3680 | 2,971967263 | 2,746192082 | 0,014 probable methylmalonate-semialdehyde dehydrogenase [acylating] mitochondrial [Crass | XP_022331028.1 |
| 49 | 3,756631422 | 3,544568849 | 0,014 myosin heavy chain isoform N [Anopheles sinensis] | KFB49247.1 |
| 12 | 3,857270689 | 3,626956434 | 0,014 tubulin alpha-1A chain [Mizuhopecten yessoensis] | XP_021370666.1 |
| 268 | 2,886887969 | 2,682676468 | 0,015 14-3-3 protein homolog 2-like [Crassostrea virginica] | XP_022343021.1 |
| 76 | 2,992121234 | 2,770492849 | 0,015 histone variant H2A.X [Mytilus galloprovincialis] | AEH58057.1 |
| 137 | 3,474746041 | 3,147817508 | 0,015 PREDICTED: peptidyl-prolyl cis-trans isomerase [Crassostrea gigas] | XP_011444269.1 |
| 233 | 2,720369268 | 2,505415611 | 0,015 PREDICTED: nucleoredoxin [Strongylocentrotus purpuratus] | XP_011682183.1 |
| 99 | 3,126761993 | 2,899738829 | 0,016 twitchin-like protein-1 [Mytilus coruscus] | AKS48140.1 |
| 105 | 3,064106025 | 2,735584039 | 0,016 flotillin-1-like isoform X1 [Mizuhopecten yessoensis] | XP_021361554.1 |
| 113 | 3,292454202 | 3,080518443 | 0,016 PREDICTED: 60S ribosomal protein L11 [Biomphalaria glabrata] | XP_013068480.1 |
| 169 | 3,020716799 | 2,388227171 | 0,017 dolichyl-diphosphooligosaccharide--protein glycosyltransferase 48 kDa subunit-like [Mizu | XP_021367901.1 |
| 271 | 2,717329052 | 2,430972595 | 0,018 hypothetical protein AM593_10055 partial [Mytilus galloprovincialis] | OPL21226.1 |
| 260 | 2,545741083 | 2,309469002 | 0,018 cytochrome c oxidase subunit IV partial [Mytilus edulis] | ACA23124.1 |
| 222 | 2,565493834 | 2,327268004 | 0,019 annexin B9-like [Crassostrea virginica] | XP_022317867.1 |
| 161 | 2,620104329 | 2,352810492 | 0,021 twitchin partial [Mytilus galloprovincialis] | BAB17763.1 |
| 111 | 3,193063225 | 3,003300028 | 0,022 small heat shock protein 22 [Mytilus galloprovincialis] | AEP02967.1 |
| 7964 | 2,839162383 | 2,517217332 | 0,023 trichohyalin-like isoform X4 [Crassostrea virginica] | XP_022318647.1 |
| 15 | 4,294007307 | 4,082692656 | 0,023 calponin-like protein [Mytilus galloprovincialis] | BAB60813.1 |
| 368 | 2,720025856 | 2,589036733 | 0,023 T-complex protein 1 subunit beta-like [Crassostrea virginica] | XP_022286485.1 |
| 225 | 1,968699847 | 0 | 0,024 PREDICTED: heterogeneous nuclear ribonucleoprotein 87F-like isoform X3 [Aplysia califor | XP_012945445.1 |
| 342 | 2,808698205 | 2,648177703 | 0,025 tektin-2-like [Crassostrea virginica] | XP_022314135.1 |
| 1149 | 3,419776302 | 3,146196286 | 0,026 PREDICTED: centlein [Crassostrea gigas] | XP_019925172.1 |
| 235 | 2,587665791 | 2,433906158 | 0,026 PREDICTED: alcohol dehydrogenase class-3-like [Lingula anatina] | XP_013411149.1 |
| 26 | 3,521384225 | 3,314636334 | 0,026 protein disulfide-isomerase A3-like [Crassostrea virginica] | XP_022329914.1 |
| 117 | 2,559659846 | 2,323211355 | 0,026 LOW QUALITY PROTEIN: annexin A4-like [Crassostrea virginica] | XP_022316814.1 |
| 249 | 3,162984532 | 3,002267821 | 0,028 uncharacterized protein ZK1073.1-like isoform X4 [Crassostrea virginica] | XP_022335676.1 |
| 102 | 2,963224744 | 2,738570733 | 0,028 twitchin [Mytilus galloprovincialis] | BAC00784.1 |
| 227 | 2,45641341 | 2,284274011 | 0,028 PREDICTED: cilia- and flagella-associated protein 52 [Crassostrea gigas] | XP_011416101.1 |
| 44 | 3,497746166 | 3,336378797 | 0,028 hypothetical protein HELRODRAFT_186143 [Helobdella robusta] | XP_009029528.1 |
| 217 | 2,565041916 | 2,379855841 | 0,029 insulin-like growth factor 2 mRNA-binding protein 2 isoform X1 [Crassostrea virginica] | XP_022305782.1 |
| 307 | 3,333337048 | 3,545860356 | 0,035 No_Hits | |
| 252 | 2,597587435 | 1,80007368 | 0,035 PREDICTED: villin-1 isoform X1 [Crassostrea gigas] | XP_011417460.1 |
| 90 | 3,241577044 | 2,928062732 | 0,035 citrate synthase mitochondrial-like [Mizuhopecten yessoensis] | XP_021357329.1 |
| 27 | 3,511154726 | 3,378470728 | 0,036 filament-like protein-2 [Mytilus coruscus] | AKS48133.1 |
| 258 | 1,974097387 | 0 | 0,037 uncharacterized protein LOC110464458 isoform X12 [Mizuhopecten yessoensis] | XP_021375365.1 |
| 144 | 3,876583641 | 3,630314254 | 0,037 No_Hits | |
| 5888 | 2,439054532 | 2,261881695 | 0,038 testis-specific gene 10 protein-like isoform X2 [Mizuhopecten yessoensis] | XP_021348266.1 |
| 229 | 2,513041968 | 2,363350566 | 0,040 Lethal(2) giant larvae protein-like 1 [Mizuhopecten yessoensis] | OWF45664.1 |
| 3693 | 1,482260274 | 0 | 0,043 clathrin heavy chain 1 isoform X1 [Mizuhopecten yessoensis] | XP_021354511.1 |
| 9 | 4,241189316 | 4,086966549 | 0,048 retrograde protein of 51 kDa-like isoform X4 [Crassostrea virginica] | XP_022320110.1 |
| 25 | 0 | 1,28591816 | 0,049 tropomyosin isoform X9 [Crassostrea virginica] | XP_022332780.1 |

*Table S3: Functional analysis and enrichment of differentially expressed proteins identified in mussel plantigrade larvae. Only the GO category Biological Process is represented in the analysis. GO = gene ontology term; term_size = number of gene sequences related to the GO term in the database; effective_domain_size = total number of gene sequences in the database.*

| term_name | term_id | term_size | effective_domain_size | adjusted_p_value__XA21 | query_size__XA21 | intersection_size__XA21 | adjusted_p_value__XA23 | query_size__XA23 | intersection_size__XA23 |
|---|---|---|---|---|---|---|---|---|---|
| organonitrogen compound biosynthetic process | GO:1901566 | 629 | 8980 | 1,17856E-05 | 70 | 20 | 8,93813E-06 | 69 | 20 |
| translation | GO:0006412 | 292 | 8980 | 0,000741043 | 70 | 12 | 8,80207E-05 | 69 | 13 |
| peptide biosynthetic process | GO:0043043 | 300 | 8980 | 0,00098223 | 70 | 12 | 0,000120158 | 69 | 13 |
| amide biosynthetic process | GO:0043604 | 312 | 8980 | 0,001474339 | 70 | 12 | 0,000188239 | 69 | 13 |
| peptide metabolic process | GO:0006518 | 319 | 8980 | 0,001852446 | 70 | 12 | 0,000242306 | 69 | 13 |
| cellular amide metabolic process | GO:0043603 | 358 | 8980 | 0,005961851 | 70 | 12 | 0,000884403 | 69 | 13 |
| small molecule metabolic process | GO:0044281 | 526 | 8980 | 0,000669304 | 70 | 16 | 0,002689155 | 69 | 15 |
| cellular component biogenesis | GO:0044085 | 349 | 8980 | 0,024340054 | 70 | 11 | 0,003936325 | 69 | 12 |
| cilium movement involved in cell motility | GO:0060294 | 10 | 8980 | 0,000231887 | 70 | 4 | 0,016823585 | 69 | 3 |
| cilium-dependent cell motility | GO:0060285 | 11 | 8980 | 0,000362252 | 70 | 4 | 0,023005128 | 69 | 3 |
| cilium or flagellum-dependent cell motility | GO:0001539 | 13 | 8980 | 0,000775683 | 70 | 4 | 0,039438064 | 69 | 3 |
| purine ribonucleoside triphosphate metabolic process | GO:0009205 | 17 | 8980 | 0,002521873 | 70 | 4 | 0,09172431 | 69 | 3 |
| purine ribonucleoside triphosphate biosynthetic process | GO:0009206 | 17 | 8980 | 0,002521873 | 70 | 4 | 0,09172431 | 69 | 3 |
| purine nucleoside triphosphate metabolic process | GO:0009144 | 17 | 8980 | 0,002521873 | 70 | 4 | 0,09172431 | 69 | 3 |
| purine nucleoside triphosphate biosynthetic process | GO:0009145 | 17 | 8980 | 0,002521873 | 70 | 4 | 0,09172431 | 69 | 3 |
| cilium movement | GO:0003341 | 18 | 8980 | 0,003223374 | 70 | 4 | 0,109464658 | 69 | 3 |
| ribonucleoside triphosphate metabolic process | GO:0009199 | 19 | 8980 | 0,004058976 | 70 | 4 | 0,129275572 | 69 | 3 |
| nucleoside triphosphate biosynthetic process | GO:0009142 | 19 | 8980 | 0,004058976 | 70 | 4 | 0,129275572 | 69 | 3 |
| ribonucleoside triphosphate biosynthetic process | GO:0009201 | 19 | 8980 | 0,004058976 | 70 | 4 | 0,129275572 | 69 | 3 |
